# Conducting an Agricultural Life Cycle Assessment: Challenges and Perspectives

**DOI:** 10.1155/2013/472431

**Published:** 2013-12-10

**Authors:** Kevin R. Caffrey, Matthew W. Veal

**Affiliations:** Biological and Agricultural Engineering, North Carolina State University, Box 7625, Raleigh, NC 27695, USA

## Abstract

Agriculture is a diverse field that produces a wide array of products vital to society. As global populations continue to grow the competition for natural resources will increase pressure on agricultural production of food, fiber, energy, and various high value by-products. With elevated concerns related to environmental impacts associated with the needs of a growing population, a life cycle assessment (LCA) framework can be used to determine areas of greatest impact and compare reduction strategies for agricultural production systems. The LCA methodology was originally developed for industrial operations but has been expanded to a wider range of fields including agriculture. There are various factors that increase the complexity of determining impacts associated with agricultural production including multiple products from a single system, regional and crop specific management techniques, temporal variations (seasonally and annually), spatial variations (multilocation production of end products), and the large quantity of nonpoint emission sources. The lack of consistent methodology of some impacts that are of major concern to agriculture (e.g., land use and water usage) increases the complexity of this analysis. This paper strives to review some of these issues and give perspective to the LCA practitioner in the field of agriculture.

## 1. Introduction

The World Bank estimates the added value from agriculture accounted for a little over 3% of the world GDP in 2012 [1]. Estimates for 2012 show that developing countries, like India, show higher portions of their GDP in agriculture at 17.4% ($317.6 billion of $1.825 trillion GDP) while developed countries, like the USA, are lower in the area of 1.1% ($172.5 billion of $15.68 trillion GDP) [2]. A basic definition of the word agriculture from Merriam-Webster is “the science, art, or practice of cultivating the soil, producing crops, and raising livestock and in varying degrees the preparation and marketing of the resulting products” [3] but this does not encompass the complexity of the field. Some of the wide varieties of practices that fall within agriculture are shown in Table 1. To a lesser degree some would consider the industrial production of agricultural materials (e.g., machinery, agrochemicals, and soil additives) and postharvest activities (e.g., grain mills, food processing, and transportation) to be included. This wide range of activities coupled with regional and crop specific cultivation methods make it difficult to succulently define agriculture.

Competition for natural resources will continue to increase globally with projected world populations set to reach 9.6 billion by 2050 [4]. Increased competition for resources required for agricultural production will lead to limited land, water, mineral nutrients, fuels, and so forth. To sustain the anticipated human population growth it will require agriculture to produce increased food, fiber, and biomass energy products within the bounds of these limited resources while reducing associated environmental impacts. The agricultural sector contributes to numerous environmental impacts including land use change, greenhouse gas (GHG) emissions, eutrophication, ecotoxicity, and some human health impacts. For example, the IPCC [5] report found that agriculture contributed 13.5% of the total GHG emissions in 2004. In 2013 the US National Oceanic and Atmospheric Administration projected record setting dead zones in the Gulf of Mexico [6] partially related to nutrient displacement from agricultural activities along the Mississippi River. To determine environmental impacts associated with agriculture the use of a life cycle assessment (LCA) framework allows the entire production chain to be analyzed for a wide variety of applications.

An LCA is a quantitative method for determination of environmental impacts across an entire supply chain. General guiding principles for this analysis can be found in ISO 14040 [7] and ISO 14044 [8]. The assessment can be split into four distinct sections: (1) goal and scope definition, (2) life cycle inventory analysis (LCI), (3) life cycle impact assessment (LCIA), and (4) life cycle interpretation. This method was originally developed for use in industrial operations but has later been adapted for a wider range of applications including agriculture. Accounting for environmental impacts associated with agriculture has some distinct challenges including the wide range of agricultural activities (Table 1) as well as spatial (e.g., dissipated emissions and differing regional conditions) and temporal (e.g., year-to-year crop rotations and seasonal fluctuations) variations inherent to the field.

There have been a number of publications that have used the LCA framework for agricultural practices. Products like oil crops [9], sugar beets [10], wheat [11], apples [12], pork [13], and milk/beef [14] have all been analyzed using an LCA framework. Primarily these studies have originated from the European Union where use of this type of analysis is more common than that in the USA. The differences in cultivation practices and climates may not allow direct comparison between regional and national LCA analyses.

It has been generally acknowledged that organic farming practices are environmentally superior to conventional but this thought is challenged through the use of an LCA framework. In the United States the US Department of Agriculture monitors and assesses farms for their organic status [15]. Tuomisto et al. [16] found that organic systems had increases in erosion and land use but reductions in nitrate leaching and eutrophication/acidification, so a clear “winner” could not be found. Taking into account the use of environmentally sensitive practices (also known as an integrated approach) while using intensive farming practices, Nemecek et al. [17] found that organic farming on the farm scale is better but on a per unit production scale the integrated process prevails. Both Hayashi [18] and Boer [19] propose that when assessing organic farming practices use of a functional unit (unit of measure, e.g., kg of product) and the product allocation procedure affects the final outcomes of the analysis.

Another consideration for agricultural production is extensive compared to intensive farming practices. “Extensive” is related to the use of additional land area with reduced material inputs while “intensive” means optimizing material inputs for greatest yields, thus utilizing reduced land surface. Nemecek et al. [20] found that extensive farming is the best form due to deceased material inputs, but reduction of different material inputs affects various impacts. Tuomisto et al. [21] found that the use of a functional unit has a large impact on the results comparing extensive versus intensive production systems. Use of functional unit and variations between environmental impacts are common in LCA analysis making it difficult to determine a “best” environmental option. Use of a normalization procedure and assessing outcomes across multiple functional units can help avoid some of these problems but bias can still exist in the results.

The objective of this paper is to promulgate the current challenges associated with conducting an LCA related to agricultural production and to provide some perspective on how to overcome these complications. This paper proceeds through the general framework on an LCA analysis giving insight into the issues and potential solutions related to each section and the analysis in its entirety. With the complex nature of agriculture today and the myriad impacts associated (e.g., land use change, eutrophication, climate change, and human health effects) the use of a comprehensive LCA analysis will help to alleviate some of the stress related to an increasing global population.

## 2. Goal and Scope Definition

The goal and scope definition sets up the basic methodology of the specific LCA to be conducted ensuring uniformity throughout analysis. This portion of the analysis is incredibly important since it sets the stage for how the entire agricultural system will be interrupted. Spending time to properly determine how the LCA analysis will occur helps decrease the time needed to address difficulties when faced with one of the many challenges associated with evaluation of agricultural activities.

### 2.1. Goal(s)

Determination of the goal(s) of the LCA will specify how the rest of the analysis is conducted. ISO [7] describes this portion as requiring four components: intended application, reason to carry out the LCA, intended audience, and if the results will be used in a comparative analysis. In the context of agriculture, there are various reasons why the use of an LCA may be warranted including voluntary standards set by the individual farm owner, encouragement from processing facilities, regulatory requirements, encouragement by a trade organization (e.g., National Cotton Council of America), and improving public perception of specific products. The scope of the analysis may be altered depending on why the LCA is being conducted and the intended audience. When used for comparative purposes, it may be possible to limit the scope to areas that will be affected by the scenario modifications, since these differences are the primary interest (e.g., when assessing various grain drying possibilities, field operations may remain static between scenarios).

### 2.2. Scope

There are various portions included in the framework of an LCA [7, 8] that are used to determine the structure of the analysis. A list of items that need to be addressed in the scope from ISO 14044 [8] are shown in Table 2. All of these requirements are important to be considered in any LCA, but with regard to agriculture system boundary, functional unit, allocation procedure, life cycle impact assessment (LCIA) methodology (and impact selection), and data quality requirements are extremely critical.

#### 2.2.1. System Boundary

A system boundary determines what will and will not be addressed in the LCA. Multiple parts of the scope need to be addressed while establishing this boundary but most importantly it needs to be in accordance with the primary goal(s) of the study. This may not be incredibly difficult in the context of industrial products with emissions being dominated by direct flows with fairly uniform output throughout the year. Environmental impacts associated with agriculture are dominated by nonpoint sources which vary temporally, both seasonally and annually.

Point source emissions are attributed to end of pipe or end of stack emissions, both aqueous and gaseous. These can be relatively easily monitored using existing equipment and in many industries records of these emissions are required for regulatory compliance. Nonpoint emissions are more difficult to quantify with sources like nitrogen volatilization from soil additives, erosion due to tillage operations, and nitrate leaching from manure handling practices. Using average or modeled results for some of these sources may be the most appropriate way to quantify them. Nonpoint emissions are widespread in agricultural operations and can account for a considerable quantity of total emissions.

Emission sources in LCA can be divided into direct and indirect. Direct sources come from the system being observed while indirect emissions stem from material inputs into these systems. An example of direct emissions in agriculture would be the engine exhaust from a tractor used in the cultivation of a field crop. Indirect sources in agriculture could include emissions related to the production of fossil fuels, soil additives, and equipment. Depending on the size of the system being investigated these may vary but an LCA cannot encompass all aspects of production. Two common designations for agricultural practices are fieldgate and farmgate. Fieldgate production goes to the point of field edge (e.g., corn in the combine) while farmgate goes to the point of material leaving the farm (e.g., corn after drying and storage). There are times that these may be the same thing but it has been shown that value-added agricultural practices (additional processing at farm) can have some significant benefits to rural development [22].

System boundary is incredibly important especially when looking at agricultural systems, due to the large amount of material processing of inputs and processing of materials past the farmgate. Cooper et al. [23] argue that the farmgate is the best location to end analysis since there are major differences in end product processing, many of which the farmer has no effect on. The impact of various system boundary sizes is evaluated by Roer et al. [24] who found that the system boundary has a tremendous impact on LCA results, as do issues with data uncertainty and data sources. When evaluating land use change comparing organic versus conventional farming practices Tuomisto et al. [21] also found that the system boundary had a significant effect on results.

If the LCA is taken beyond the farmgate, additional analyses may include end product processes such as food processing, transportation, and postharvest handling. Generally, it is preferred to split these processes into modules to simplify analysis (treat each system individually). This becomes increasingly difficult when trying to deal with the impacts associated with employees [25], retail operations [26], on-farm energy production [27], value-added agricultural practices [22], and areas of the farm not associated with crop production (such as timber stands or agritourism). Ruviaro et al. [28] strongly suggest that processing after the farmgate can have a major impact on results complicating the overall analysis. When looking at the entire production chain Bevilacqua et al. [29] found that at home cooking of pasta used the most energy out of the entire production chain. Essentially, the energy required to cook the pasta is more significant in the life cycle of the production than energy consumed to grow the wheat or process it into pasta. Using this entire supply chain approach correctly asserts that at home energy reductions are the most impactful but if the goal is to reduce on-farm energy usage other important reduction strategies may be overlooked.

#### 2.2.2. Functional Unit and Allocation Procedure

The functional unit is the quantifiable value associated with the function of the system (e.g., function: corn production, unit: kg/ha). Depending on the system being analyzed and the goals of the LCA defining functional units can become complex for agricultural systems. From the perspective of a whole farm enterprise, it is easy to identify multiple products whose production may vary annually depending on crop rotation schedules and yield variations. To address these challenges, an allocation procedure is required. The allocation procedure is the operation of dividing the impacts between the various products coming from an agricultural operation. This is an area where bias that may show one product with reduced impacts while inflating another can be introduced.

If the functional unit is subdivided between specific crops defining functional units may still be difficult with multiple products possible from a singular source (e.g., milk and beef from dairy operations). A field scale analysis can be made but this needs to take into account winter crops, either winter cover (i.e., crops to promote soil health) or economic products (e.g., winter wheat or canola), and variations in annual production between crop rotations. The allocation of functional units should also take into account products produced for on-farm usage such as bioenergy crops, organic soil additives, and animal feed. There are various methods of allocation that may be appropriate when taking primary and by-products of the system into account, including weight, economic value, system expansion, by-products accounted for by displacement, or the entire farm considered as a single unit. System expansion refers to modularizing the assessment to subdivide operations into their own analyses; this adds complexity for shared equipment and facilities (e.g., greenhouses for tobacco plant production are also used for vegetable production in nontobacco producing months). Displacement is the process of accounting for by-products by determining what materials will be displaced by their use; this can also be applied to primary products if applicable within the confines of the analysis (e.g., use of crop residues for greenhouse heating instead of fuel oil).

Use of a functional unit is tied directly to the goals of the analysis and the audience that the LCA will address. If analysis is requested by downstream operations for use as internal industrial purposes the production of a specific product may be the interest. When used for public perception reasons the entire farm unit may be addressed but the use of a third party to conduct a critical review of the LCA to limit bias is strongly suggested. A number of studies [11, 18, 21, 30, 31] found that the definition of functional unit was a major factor in results. Eady et al. [32] found that the complexity of agricultural systems and the multiple products associated makes it incredibly difficult to model these systems (e.g., a sheep operation produces wool, animals for slaughter, stud rams, surplus animals for other farmers, manure for use as a soil additive, and various crops on arable land).

#### 2.2.3. Life Cycle Impact Assessment (LCIA) Methodology

Choosing the proper methodology and specific impacts of interest can be connected to the goals of the analysis. Some studies are primarily focused on GHG emission reduction strategies within the confines of an LCA framework. Other analyses may be more concerned with eutrophication potentials that result from local or regional disturbances. Some may want to determine their current environmental impacts across a series of metrics to determine future mitigation strategies. It is important to note that it may not be possible to reduce all environmental impacts and some sort of concession must be made.

There are commonly two forms of impacts assessments, midpoint and endpoint, though there are a number of reference units and methodologies within each. Midpoint procedures use scientifically verifiable results [33] to standardize emission to a specific reference (e.g., nitrous oxide to CO_2_ equivalents). Endpoint impacts strive to connect emissions with observable effects (e.g., increase in hypoxia from soil additive displacement). Though the end results of our impacts on the environment are of greater concern, these are difficult to assess due to local conditions, management practices, and temporal variations. For example nitrate leaching from soil additives will be affected by local hydrology, use of best management practices (BMPs), and time of the year (year-to-year weather variations are equally important). When assessing heavy metal impacts on human health Pizzol et al. [34] found that the use of different LCIA methodologies made it difficult to draw conclusions between the results. This difficulty arose from the specific metals listed and characterization factor calculations used in each of the methodologies [34]. Use of an improper LCIA methodology may result in inflated results in one or more of the impact factors, suggesting reduction strategies that may not be required for specific locations, emissions, or discharge schedules. With some impacts covering a diverse area (e.g., land use change and biodiversity) it is important that mitigation strategies are aimed at the specific goals of the study not at the calculated metric (e.g., if the goal is to increase habitat for a local species optimizing the biodiversity score may not do this). Accounting for an expanded host of environmental impacts, beyond those specifically of interest, may confuse the results requiring concessions between reductions and increases in impacts. For example, to significantly reduce eutrophication may require increased land use and increase human health impacts in some areas (e.g., a treatment system can reduce eutrophication potential but it takes land and may produce ammonia and other inhalation irritants).

#### 2.2.4. Data Quality Requirements

Determining the level of data quality required for the specific analysis will affect data retrieval efforts. Data from standardized databases (either nationally or regionally based), collection of empirical field data, data resolution, and the frequency of observations are data quality issues that must be considered. Regional databases can be strengthened by taking limited field observations from specific points to ensure database quality (e.g., testing tobacco curing stack emissions compared with database values). Operational data also needs to be either determined from on-farm processes or standardized information sources (e.g., actual versus literature fuel usage for tractors). Level of complexity for nonpoint source models should be taken into account when determining the level of data quality (e.g., use of a highly complex model with inaccurate or incomplete data may yield unrealistic results and vice versa). Since seasonal variations and weather can have a profound effect on agricultural practices, it will be necessary to use multiple years' worth of data to generate a proper average. Depending on the goals of the study this may mean using information from a “good,” “average”, or “poor” year of operations, again either using actual or standard data for operations. Most likely there will be some data that is difficult to use from regional sources because data is incomplete, outdated, or unsatisfactory. Though use of regional or local values may relate to the goal(s) of the study, data from a larger demographic (e.g., national) or other geographic locations may be preferable.

The use of large well maintained databases and government sources is commonly employed for LCA analysis but these still have some data quality issues associated. Cooper et al. [35] found a large amount of sampling error associated with USDA ERS crop unit process data because of limited samples, timeframes, and changes in operations over time. Uncertainty related to GHG production from farms is shown in Gibbons et al. [36] from both a spatial and a temporal perspective. Differences in crop management practices also have an effect on crop yields, some of which show soil quality benefits in later years after application [37]. Understanding the level and areas of uncertainty in the analysis will assist with interpreting results and need to be addressed with the final report.

## 3. Life Cycle Inventory Analysis (LCI)

Completion of the life cycle inventory analysis collects and calculates emissions data. These values take into account the system boundary and allocation procedure as described in the scope. The data can be divided into various categories such as energy, raw materials, products, waste, air emissions, water emissions, and discharges to soil. After the data has been collected the inventory values are calculated with allocation procedure and functional unit in mind.

Some emissions related to agriculture are direct point sources similar to industrial processes (e.g., corn grain drier stack emissions and a lagoon overflow pipe). Indirect emissions relate to upstream production processes that may be vital depending on the goal(s) of the analysis (e.g., alternative soil nutrient sources or agro-chemical usage). Many of the most important emissions in agriculture are either nonpoint or indirect (Table 3) but some that are especially complex in the context of the LCA framework are land use change, water usage, soil additives, and livestock production systems.

### 3.1. Land Use Change

With increasing global populations limited land availability will become a major issue in the 21st century [38]. There are generally two categories of land use change, direct and indirect, and these have differing implications regionally and globally. Direct land use change relates to modification of a land parcel (e.g., a farmer changing a meadow into a corn field) while indirect land use change is the effect of modified land use on other areas (e.g., reduced corn exports from the United States may require deforestation in other countries to meet local feed demands). Currently most LCA methodologies use a metric of arable land use (m^2^) but an expanded definition is needed to take into account the many related aspects.

Indirect land use changes have been shown to have major effects on production of nonfood agriculture (bioenergy feedstocks) by Searchinger et al. [39] and Fargione et al. [40]. Brazil and some areas of Africa are shown to have some of the largest increases in agriculture and subsequently land use change [41]. Ruviaro et al. [28] mention that though reducing environmental impacts is important in Brazil so is facing the need to feed an increasing global population. It is incredibly difficult to give a direct impact to indirect land use change without taking a number of assumptions that may introduce bias into results. Commonly the system boundary set for the LCA analysis is too narrow to include many of these effects. The scope of the LCA can be expanded to take into account indirect land use change but this requires understanding of the specific impacts associated with the land disturbance (e.g., where it will occur, what will change, what supplemental crops will be planted, and what crop management techniques will be used for the region).

A number of studies have shown how direct land use change can affect carbon sequestration [42–44]. Though even the determination of impacts related to direct land use change is not simple, there are some metrics that are possible to assess. Impacts such as biodiversity and aesthetics as detailed by Haas et al. [30] are difficult to determine but the proposed metrics of crop management and soil quality are more realistically quantified. To reduce GHG emissions Kulak et al. [45] found positive benefits associated with the use of urban agriculture, showing a positive impact of land use change though positive impacts are commonly ignored in the LCA framework. It is important to remember that the land owner ultimately has the final determination in what they do with their own property, but it is important to understand the impacts associated with modifications of land parcels.

#### 3.1.1. Crop Management (Tillage)

Tillage is the mechanical manipulation of soil particles and plays a key role in soil preparation for crop production. Tillage activities and intensity vary greatly depending on region, crop, and management practices. The use of a minimum tillage strategy is generally accepted across the United States since it improves soil stability, moisture retention, and fuel costs and reduces equipment usage. While no till is a proven strategy it has limitations, and depending on the soil condition, the specific crop, and past use of the field more intense tillage operations may be required. With these increased tillage operations, two environmental impacts will see significant changes: GHG emissions and erosion control.

Intensive tillage operations reduce carbon sequestration of soils and increases fossil fuel usage in equipment [46, 47]. Carbon sequestration is a useful tool for reducing atmospheric CO_2_ levels and West and Marland [48] argue for the proper use of carbon sequestration in the analysis of carbon emissions from different tillage operations, some of which show positive benefits. The effects of tillage on soil include biological, chemical, and physical property changes and these are further related to ability of the soil to sequester carbon [49]. Being realistic with proposed management changes in context of an LCA analysis is important when related to tillage operations (e.g., proposing a no-till option for a crop species and/or soil type where it is not appropriate will undermine the validity of results).

In order to preserve high quality top soil, many farms place importance on management practices that focus on reducing soil erosion. In addition to potentially harming crop production, soil erosion is a major environmental issue that can lead to hydrologic changes, ecosystem disruption, inhalation concerns, and settlement in lakes and dams. In the United States, soil erosion is mitigated through the use of BMPs which include riparian zones, surface residue retention, and hillside meadow strips. Though most current soil erosion control is focused on water-born concerns, the Dust Bowl of the 1930s [50] showed farmers in the United States that wind erosion is a major cause of soil loss. Modified crop management practices will have an effect on soil erosion and the subsequent environmental impacts. Pimentel et al. [51] found that erosion had the potential to threaten world food production, which with increased populations today is an even greater issue. An editorial by Glanz [52] about work done by Pimentel et al. [53] discussed the excessive cost that erosion is causing to the United States. Soil rates of production are shown to be one to two orders of magnitude greater under native vegetation compared to conventionally plowed agricultural fields [54]. Soil erosion is a major concern for agricultural practices so mitigation strategies need to be addressed correctly in an LCA framework. Some of these systems may introduce net environmental benefits such as the use of riparian zones that will increase biodiversity and reduce transport of other harmful water based emissions.

#### 3.1.2. Soil Quality

The general loss of soil through erosion is a major concern but changes in soil quality can also be affected by agricultural practices. Soil quality is a rather general term that can be interpreted in multiple ways and therefore has many different metrics for determination. Issues related to soil quality include salinization, compaction, chemical and soil additives, desertification, and changes in soil organic matter.

In areas with fragile soil structures salinization can have major implications on soil quality and the ability for an area to remain in agricultural production. Increased salinity can be attributed to poor irrigation practices that lead to excessive water logging of the soil. Feitz & Lundie [55] argue for a single parameter related to erosion, acidification, soil structure decline, and salinity since they are interrelated in areas where salinity is a major concern. However, from a life cycle perspective determination of these factors over long term management periods can be extremely difficult without extensive long term in-field observations.

Soil compaction can be a major issue in regions where managed crop production is regularly used, like in the United States. Heavy equipment traffic and reduced cover can have substantial effects on compaction. A methodology is developed by Garrigues et al. [56] using equipment traffic patterns and weights instead of in-field measurements, which is extremely useful in place of costly field testing. It may be useful to take limited in-field measurements to ensure that modeled data is compatible with field observations.

Chemical and soil additives can both affect the soil quality depending on management practices and weather conditions. Use of wood ash in place of a liming agent has the potential to add toxic heavy metals to the soil which may affect plant growth [57], which needs to be accounted for in the LCA. Though it is common to focus on human and ecosystem toxicity with chemical application [58], the potential for soil contamination exists as an additional impact. Soil contamination may result in human exposure via plant uptake which Fantke et al. [59] found to require a substantial number of inputs for proper determination, therefore creating a high level of uncertainty of results.

The soil organic matter is an important component of the soil that has substantial effect on carbon sequestration [60] and is relatively simple to measure in the field. Cowell & Clift [61] argue for the use of soil organic layer depth and soil compaction to determine soil quality. This is also an area that is highly influenced by tillage operations and erosion. Proper soil management can also result in an increased organic layer that needs to somehow be addressed as a net benefit [62]. Depth of the soil organic layer will vary spatially in a single field due to various components (e.g., slope, cover, and hydrologic conditions) so proper measurements or averaged values from modeling approaches need to be taken into account for accuracy of results.

Desertification is the process of arable land being converted to something more similar to a savannah through climatic changes and management decisions. Núñez et al. [63] argue for an LCA metric related to desertification using conditions related to aridity, erosion, aquifer, and fire risk to make a determination. This process was used by Civit et al. [64] and was found to be useful in dry land areas. With expanding desert regions in many developing countries this is especially problematic. Metrics like desertification have regional implications that may not need to be addressed in areas that are less susceptible, though the inclusion of indirect land use change may require the use of this metric to some degree.

### 3.2. Water Use

Though water covers the vast majority of land area around the world, potable water sources are in limited supply. According to USDA 80% of the United States water consumption is from agricultural operations which can spike at levels above 90% in some western states [65]. Prominently in the United States the Ogallala aquifer has been drastically reduced from agricultural activities in the high plains causing fear of reduced arable land availability in this highly agriculturally productive area [66]. Currently most LCA methodologies either do not consider water or are accounted for by use alone (m^3^). There are local and regional effects related to water usage that need to be taken into account (e.g., water usage in Arizona affects water rights in Mexican states). Though the spatial considerations are important, temporal variations exist seasonally and annually, such as drought years. Bartl et al. [67] argue that the source of the water has a major impact on the environmental effects related (specifically biodiversity). There are various sources of water that will vary in their effects per region including shallow aquifers, deep aquifers, river, tap, and farm ponds. Agricultural practices can have major effects on water quality that impact other people's water sources downstream. Though current LCA work is limited on water issues, Tendall et al. [68] found that some sort of credit needs to be applied for practices that increase water quality. Depending on the scope of the LCA analysis water usage issues may be an important aspect but the complex nature of implications related to use may need to be constrained (e.g., accounting for species diversity in the future due to changes in aquifer recharge may be too broad of an area to accurately account for).

### 3.3. Soil Additives

Many current agricultural systems for crop production make tremendous use of soil additives to promote plant growth while returning important nutrients to the soil. Soil testing is recommended prior to any treatment to limit application rates to the optimal levels for the specific soil conditions and crop to be cultivated. Three primary nutrients are nitrogen, phosphorous, and potassium (NPK) but macronutrients also include calcium, magnesium, and sulfur. A series of micronutrients may also be required depending on need. Soil pH can be important for some crops and liming agents may be needed to adjust soil pH. In the United States there are BMPs for application of soil additives to limit nutrient leaching and volatilization. But such BMPs cannot always be rigidly followed (e.g., weather conditions may limit application times). Crop rotation strategies are used to replenish some of the soil nutrients (e.g., planting legumes with nitrogen fixing capacity) but these are not able to replenish all nutrients for yield optimization. Both organic (manure and municipal solid waste) and inorganic (man-made) sources of soil additives are used but the farmer may have limited material selection decisions based on variations in nutrient production locations, manure management strategies, crop requirements, and economics. Municipal solid waste is increasingly being used for land application in the European Union due to landfill regulations [69–72] but commonly needs to be supplemented with other products. For pH management quicklime is primarily used but wood ash has been shown to be a significant liming agent and source of some required nutrients [73]. The two primary direct impacts, other than equipment usage related to soil additive application, are leachate and volatilization. Indirect emissions (including nutrient production and transportation) should also be taken into account for an LCA. It is common for LCA studies to evaluate both leachate and volatilization of soil nutrients using estimations from many different models. A whole host of models have been developed to determine emissions from these processes requiring different levels of data about the system. Use of specialized models that require a high level of data input may be too specialized and difficult to acquire local data. Incomplete or inadequate data sources used in complex models may output inferior results to the use of a simplified model with straightforward inputs. It is similar to the use of limited field data in place of modeled results.

#### 3.3.1. Leachate

The major environmental impact related to leachate of soil additives is eutrophication (i.e., nutrient addition to natural water bodies), which then has the potential to cause algal blooms resulting in zones of hypoxia (i.e., reduced dissolved oxygen levels). Reductions in leachate are possible through use of BMPs, changes in crop management, crop species, and reduced nutrient use. Brentrup et al. [10] used a method developed by the German Soil Science Society to determine nitrate leaching. A model developed by Johnson & Parker [74] was created specifically for a region in northern Virginia. Other specific leachate models have been developed for different regions with varying levels of complexity. The results of these models can then be included in the inventory section of the LCA to take into account this important agricultural impact.

#### 3.3.2. Volatilization

Many impacts are possible from the volatilization of soil additives but the primary focus has been on nitrous oxide and ammonia emissions from man-made nitrogen fertilizers. Organic fertilizers, such as manure from livestock, can also have emissions of ammonia, methane, and other chemical compounds. IPCC [75] uses a simplified methodology for determination of volatilization of nitrous oxide, 1% of applied nitrogen and crop residues. Brentrup et al. [10] use emission factors from a more complex model (the ECETOC model) to determine nitrogen volatilization. Nitrous oxide emissions from soil additives and agricultural practices specifically related to biofuels production were reviewed by Ogle et al. [76]. Nitrous oxide is related to an increase in global warming potentials, and methane has impacts on global warming and smog production, while ammonia causes acidification and eutrophication through deposition. Neglecting these emission sources may alter results and subsequent mitigation strategies.

### 3.4. Livestock

As quality of life increases in developing countries, per capita meat consumption increases significantly [77]. Couple growing meat consumption by individuals with global population growth and the production of livestock products will need to be significantly higher in coming years. With limited land availability as well as competition for other agricultural sectors (e.g., food, fiber, energy, and various high value by-product markets), livestock production is increasingly moving towards intensive production processes. Confined animal feeding operations (CAFOs) have become the standard for some livestock operations regionally (e.g., hog farming on the southern seaboard of the United States). CAFOs concentrate waste products and require large volumes of material inputs from outside sources. Differences in production systems occur regionally between animal species and management practices. The two main sources of direct emissions related to operations are methane emissions from enteric fermentation and manure handling systems.

For some operations this includes the movement of livestock between age classes such as in industrial production of broiler and hog farming. Depending on the scope of analysis transportation between these systems may be included. Large regional and farm variations in management practices exist between livestock production systems that may need to be taken into account. There are a number of publications that investigate the difference between organic and conventional livestock systems using an LCA framework [78–80]. These commonly differ in determination of the “best” environmental option because of the complex nature of the various environmental impacts.

#### 3.4.1. Enteric Fermentation

Methane produced from enteric fermentation in ruminants (e.g., cows, sheep, and goats) is considered a major cause of GHG production globally [81]. This emission is related to digestion in the forestomach of ruminants [82] during the natural process of converting grasses to digestible carbohydrates. Bonesmo et al. [83] found that enteric fermentation and manure handling were the major issues related to livestock production, with nutrient volatilization for feed production also being a major contributor. In Canada 8% of the total GHG production is related to agriculture and 33% of this is related to enteric fermentation [84].

Potential reductions in enteric emissions have been proposed ranging from increased feed conversion efficiency and animal husbandry, to various antibiotic or biological agents [83, 85]. There are considerations from each of these mitigation procedures that may limit their applicability including required additional land, increases in material requirements leading to extended transportation, and economic considerations. There are also gaseous scrubbing methods with confined operations but these can be extremely costly and only affect the livestock at limited times (i.e., when they are in the collection area). Though there are increased environmental impacts associated with ruminates it is important to consider that they convert grass and other inedible, or waste, materials into food and various essential products for society.

#### 3.4.2. Manure Handling

Waste handling from livestock operations depends on the management practices employed at each farm. Extensive production systems spread manure in areas the animals graze and fields that provide onsite feed materials. This requires use of BMPs to keep waste out of water systems and decomposition is assumed to happen naturally. CAFOs concentrate waste into single locations, commonly in lagoons which reduce nutrients and organic loads though microbial decomposition, followed by land application for use as a fertilizer in crop production. Regulations and BMPs are used to reduce the potential environmental impacts in the United States.

Reductions in environmental impacts are possible through different manure management systems but properly accounting for the emissions from each stage is important. Effluent lagoons are already used to reduce nutrient concentrations and organic loading but emissions from these include carbon dioxide, methane, and hydrogen sulfide. Covering these lagoons allows the methane to be burnt off or collected, but this practice is relatively expensive and provides the farmer with marginal returns. Sandars et al. [86] found that there were considerable changes between impacts from manure management systems and depending on the metric of interest different systems could be considered optimal. Land application of the material increases the potential for leachate emissions but can displace other soil additives for crop production.

#### 3.4.3. Aquaculture

Generally the area of livestock is thought to include ruminants (e.g., cattle, sheep, and goats), poultry (e.g., chicken, and turkey), swine, and possibly equine (e.g., horse, and pony) though these are focused primarily on leisure instead of food production. Aquaculture is a growing industry with a 7.5% growth in production from 2009 to 2010 and a projected additional increase of 25% from 2012 to 2021 [87]. As global food demands increase world fisheries cannot keep up with increasing consumption resulting in the need for high intensity aquacultural production. Increased fears related to changes in global fisheries from global climate change create an additional need for sustainable aquaculture practices [88]. Henriksson et al. [89] propose the use of additional indicators including seafloor disturbance, biotic resource depletion, and loss of biodiversity when comparing aquaculture to regular fisheries catch. There are substantial ways of reducing some of the impacts associated with this practice such as the use of recirculating systems instead of flow through [90]. These recirculating systems were designed for use in areas without sufficient water sources for flow through systems but require considerably more equipment, instrumentation, and energy to operate. All of these are areas that can be addressed with an LCA framework but regional production practices need to be taken into account.

## 4. Life Cycle Impact Assessment (LCIA)

Computation of impacts associated with the values determined in the LCI requires the use of predefined impact factors that follow the procedure decided in the scope of the analysis. Use of the specific impact system can have a major effect on outputs [34]. These calculated impacts do not always reflect the actual impacts from the system. There are a number of temporal and spatial effects on determining the actual impacts of a system, which are commonly of more interest but incredibly difficult to accurately determine. Temporal variations arise from the difference in operations in agriculture over time and between years. Considering spatial considerations takes into account where the impacts are happening and the difference between local, regional, and global impacts. There are various different impacts associated with agricultural activities but some selected impacts of interest are listed in Table 4.

Optional procedures include normalization (grouping and weighting) of impact factors to standardize results and analysis of data quality. Normalization looks for inconsistencies, prepares the results for grouping or weighting, and allows the impacts to be compared at a local or a regional level. Grouping of impacts puts them in hierarchal order or by nominal associations (e.g., regional or as inputs/outputs). Addition of a weighting factor to the results allows for conversion to a single (or multiple) impact score(s) for easier comparison between scenarios. Bovea & Gallardo [91] found that the use of different normalization procedures affected which scenario was considered the “best” environmentally. These optional procedures can be incredibly helpful in interpreting results but need to be used with caution. Reporting of results before normalization in addition to these modified values is recommended for clarity purposes.

## 5. Life Cycle Interpretation

The interpretation phase of an LCA occurs continually throughout the assessment process and is used for reporting of results. This ensures that the inventory and impact stages are consistent with the goal(s) and scope of the LCA. It also includes any significant issues for the analysis, potentially some sort of sensitivity analysis, and conclusions of the study. Determining the completeness of the analysis, study limitations, and any associated problems observed during the analysis assist in the completeness and value of the assessment. Agricultural systems have many areas of data uncertainty and the significant variations between operations may hinder the use of generalized data. Some amount of actual system analysis is preferred to minimally ensure that database values are consistent with actual observations.

### 5.1. Beyond Life Cycle Assessment (LCA)

An LCA analysis is primarily concerned with environmental impacts but other areas such as economics, energy usage, and society concerns may need to be evaluated for a given system. Farmers produce products to make money so economics needs to be a fundamental consideration when comparing systems and determining mitigation strategies. When environmental considerations can be combined with some sort of economic incentive adoption of some of these practices is more accepted, as opposed to mandating changes through regulations. Glithero et al. [27] use an input output method to look at both economic and environmental impacts on bioenergy production systems. Adding economic considerations to compare mitigation strategies will force decisions based on both factors (e.g., is it worthwhile to reduce GHG emissions by half if end product cost needs to be doubled).

Use of energy on farm is another major concern due to the limited availability of natural resources globally. Energy conservation of fossil and biologically derived fuel sources should be considered whenever economically viable. Kimming et al. [92] investigated the possibility of self-sufficient organic farming through the use of agricultural residues on farm. This can also apply to residues that are transported off-site, such as corn stover, which then may affect soil quality and erosion issues depending on quantities harvested. Production of dedicated energy crops (e.g., switchgrass) would need to be addressed in context of the multiple production years from a single planting, though some impacts are related to the initial establishment years.

Society concerns related to agriculture are far reaching, including a stable and safe food supply, land aesthetics concerns, odor from manure or other management practices, and roadway material loss (e.g., corn from grain cart or chicken feathers from transport). Animal welfare issues are of high concern for some people with mixed results between LCA analyses of organic production systems [19, 93]. Trying to quantify societal concerns is relatively difficult and usually some sort of BMP is established (e.g., minimum distance of effluent spray fields from habitation).

Use of GMOs has become common in United States agriculture with 90% of corn, 90% of upland cotton, and 93% of soybeans planted in 2013 having some sort of genetic modification [94]. Strange et al. [95] used an LCA framework to show that the use of nutrient use-efficient canola shows reduced impacts compared to the conventional seed stock. Many environmental groups have argued against the use of GMOs for fear of genetic drift [96, 97] though for food safety purposes this is highly regulated by the US FDA [98]. Trying to compare the benefits versus the potential impacts is incredibly difficult especially in the context of increasing global populations and limitations on arable land for crop production. This also comes into account with places like the European Union importing the majority of their soybean meal from Brazil due to the United States use of GMO crops; this is also being seen with exports of dried distillers grains.

Safety issues related both to on-farm operations and to the products produced (i.e., food safety) are extremely important and need to be addressed for any comparative study. Reducing environmental concerns, even if they deal with human health, should not increase safety concerns related to the production system. This can be seen for some hog farm operations where different age groups are transported to different areas to reduce disease transmission. A reduction in GHG production is possible by removing the transportation but other management strategies need to be considered to decrease the potential for disease transmission.

## 6. Conclusion

The agricultural sector is incredibly diverse producing a large number of products and services vital to mankind. Variations exist globally, regionally, and locally in management practices that make it difficult for a general LCA to be conducted on agricultural products. Some of the major issues are related to the use of natural resources, land use change, livestock production systems, soil additives, and management strategies. Other issues that need to be addressed outside of the LCA framework are economics, energy usage, and societal concerns related to agriculture. Many of the environmental impacts associated with agriculture would be minimized if everyone was a celibate raw food vegetarian, but this is not a feasible solution for society. Growing populations will only increase the pressures related to limited natural resources increasing the need for agriculture to provide food, fiber, energy, and various high value by-products. The use of an LCA framework to determine areas of greatest impact and compare reduction strategies for agricultural operations is a feasible strategy for reducing environmental impacts in the face of increased global demand.

## Figures and Tables

**Table 1:** Agricultural categories and associated Fieldgate products.

Agricultural category	Select Fieldgate products
Agronomic crops	Corn, soybean, wheat
Fiber	Cotton, hemp, straw
Forestry	Pulp, sawtimber
Horticultural crops	Tomato, lettuce, herbs
Aquaculture	Fish, seafood, algae
Livestock	Cattle, poultry, swine
Ornamentals	Turf, flowers, succulents
Orchard	Tree fruit, christmas trees
Hay and forage	Silages, alfalfa, hay
Other cash crops	Tobacco, tea, coffee, cocoa

**Table 2:** Scope requirements of an LCA [8].

List of LCA scope requirements		
Product system	Function of system(s)	Functional unit
System boundary	Allocation procedure	Limitations
Interpretation method	Data requirements	Assumptions
Value choices and optional elements	LCIA methodology (and impact selection)	Data quality requirements
Type of critical review, if any	Type and format of study	

**Table 3:** Selected life cycle inventory considerations for agriculture.

Direct sources	Indirect sources
Nutrient volatilization/leachate	Soil nutrient manufacture
Direct land use change	Indirect land use change
Livestock handling	Feed production
Fuel combustion	Fuel manufacture
Soil quality/tillage	Equipment production
Agrochemical use	Agro-chemical manufacture

**Table 4:** Selected environmental impacts associated with agriculture.

Environmental impacts	Potential sources
Global warming	Fuel combustion, livestock, nutrient volatilization
Eutrophication	Nutrient leachate, ammonia deposition, nutrient manufacture
Acidification	Livestock waste, intensive crop management
Smog	Fuel combustion, ammonia volatilization, equipment manufacture
Biodiversity loss	Land use change, agro-chemical usage
Fossil fuel depletion	Fuel combustion, material inputs, equipment manufacture
Human health	Agro-chemical usage, fuel combustion, ammonia volatilization, overall pollution, GMOs
